# Steroid withdrawal after renal transplantation: a retrospective cohort study

**DOI:** 10.1186/s12916-016-0772-6

**Published:** 2017-01-12

**Authors:** Maria C. Haller, Michael Kammer, Alexander Kainz, Heather J. Baer, Georg Heinze, Rainer Oberbauer

**Affiliations:** 1 Center for Medical Statistics, Informatics and Intelligent Systems (CeMSIIS), Section for Clinical Biometrics, Medical University of Vienna, Spitalgasse 23, Vienna, 1090 Austria; 2 Department for Internal Medicine III, Nephrology and Hypertension Diseases, Transplantation Medicine and Rheumatology, Krankenhaus Elisabethinen, Linz, Austria; 3 Methods Support Team ERBP, Ghent University Hospital, Ghent, Belgium; 4 Department of Nephrology, Medical University of Vienna, Vienna, Austria; 5 Austrian Dialysis and Transplant Registry, Kematen, Austria; 6 Division of General Internal Medicine and Primary Care, Brigham and Women’s Hospital, 1620 Tremont Street, Boston, MA 02120 USA; 7 Department of Medicine, Harvard Medical School, Boston, MA USA; 8 Department of Epidemiology, Harvard T.H. Chan School of Public Health, Boston, MA USA; 9 Department of Nephrology and Dialysis, Medical University of Vienna, University Clinic for Internal Medicine III, Währinger Gürtel 18-20, Vienna, 1090 Austria

**Keywords:** Corticosteroids, Steroid withdrawal, Steroid maintenance, Immunosuppression, Kidney transplantation, Graft loss

## Abstract

**Background:**

Immunosuppressive regimens in renal transplantation frequently contain corticosteroids, but many centers withdraw steroids as a consequence of unwanted side effects of steroids. The optimal timing to withdraw steroids after transplantation, however, remains unclear. The aim of this study was to determine an optimal time point following kidney transplantation that is associated with reduced mortality without jeopardizing the allograft to allow safe discontinuation of steroids.

**Methods:**

We conducted a retrospective cohort study and computed a concatenated landmark-stratified Cox supermodel to estimate hazard ratios and 95% confidence intervals for mortality and graft loss using dynamic propensity score matching to adjust for confounding by indication.

**Results:**

A total of 6070 first kidney transplant recipients in the Austrian Dialysis and Transplant Registry who were transplanted between 1990 and 2012 were evaluated and classified according to steroid treatment status throughout follow-up after kidney transplantation; 2142 patients were withdrawn from steroids during the study period. Overall, 1131 patients lost their graft and 821 patients in the study cohort died. Steroid withdrawal within 18 months after transplantation was associated with an increased rate of graft loss compared to steroid maintenance during that time (6 months after transplantation: HR = 1.8; 95% CI, 1.3 to 2.6; 18 months after transplantation: HR = 1.3; 95% CI, 1.1 to 1.6; 24 months after transplantation: HR = 1.2; 95% CI, 0.9 to 1.5), while mortality was not different between groups.

**Conclusions:**

Our findings suggest that steroid withdrawal after anti-IL-2 induction in the first 18 months after transplantation is associated with an increased risk of allograft loss.

**Electronic supplementary material:**

The online version of this article (doi:10.1186/s12916-016-0772-6) contains supplementary material, which is available to authorized users.

## Background

Kidney transplantation is the most cost-effective treatment option for eligible patients with end-stage renal disease since transplantation is superior in terms of quality and quantity of life whilst being less costly compared to long-term dialysis treatment [1–6]. However, choosing the appropriate immunosuppressive treatment strategy is a key decision for sustained allograft function. Despite the development of novel immunosuppressants in recent years, serious side effects, such as increased cardiovascular risk, impaired immune system detection of cancer cells and antiviral defense, still result from long-term intake of immunosuppressive drugs. Therefore, various strategies to reduce immunosuppression are being investigated with the aim to alleviate toxicity associated with this treatment [7–11].

Maintenance immunosuppression usually consists of three components, one of which is frequently a corticosteroid. Steroids are effective in preventing acute rejection, which is one of the main risk factors for reduced long-term graft survival when not appropriately diagnosed and treated [12, 13]. Although triple immunosuppression allows lower steroid doses, the disadvantageous association of steroids with weight gain, hyperlipidemia, high blood pressure, impaired glucose metabolism, and osteoporosis persists [14–21]. Consequently, several randomized trials have been performed to investigate the efficacy of steroid withdrawal after kidney transplantation. However, clinical trials can only investigate withdrawal at a specific time point, which likely may not be the optimal choice. While earlier meta-analyses of these trials reported an increased rate of acute rejections and graft loss after steroid withdrawal compared with steroid maintenance, more recent meta-analyses included trials conducted with newer immunosuppressants and found no difference in graft loss [10, 22–24]. Although the increased risk of acute rejection persisted, subgroup analyses indicated that contemporary immunosuppression reduced the risk of acute rejection, and steroid withdrawal 3–6 months following kidney transplantation was suggested. In contradiction, current clinical practice guidelines discourage steroid withdrawal beyond the first week after engraftment [25]. In view of this inconsistency of recommendations, steroid withdrawal is managed differently in clinical practice and there is no consensus on the optimal timing for steroid withdrawal after kidney transplantation. Likewise, long-term outcomes after steroid withdrawal remain uncertain to date due to the limited follow-up in rather small randomized trials [11].

The aim of the present study was to evaluate long-term outcomes on graft and patient survival following steroid withdrawal compared to steroid maintenance at various points in time after kidney transplantation in order to determine optimal timing for steroid withdrawal in kidney transplant recipients. Our hypothesis was that discontinuation of steroid treatment after a ‘certain’ treatment duration could improve patient survival through a reduction in toxicity associated with prolonged steroid maintenance without jeopardizing allograft survival.

## Methods

### Study design and data sources

We conducted a retrospective open cohort study to investigate the effect of steroid withdrawal at numerous points in time following kidney transplantation on patient and graft survival using data from three sources: the OEstereichische (Austrian) Dialysis and Transplant Registry (OEDTR), the EUROTRANSPLANT database, and the Vienna Kidney Biopsy Registry, as previously done by our group [26, 27]. The OEDTR was established by the Austrian Society of Nephrology in 1970 and has almost complete follow-up – only 0.6% of all Austrian residents on renal replacement therapy have been lost since 1990. The OEDTR contains thoroughly extracted data from the original medical records in which the original data was assessed at the time of the follow-up visit by the responsible physician [28]. Data provided by the OEDTR included recipient age and sex, date of transplantation, primary renal diagnosis, the presence of comorbidities at transplantation and annually throughout follow-up, panel reactive antibodies, patient and graft survival, and immunosuppression. Use of immunosuppressive medication was reported quarterly in the first year after transplantation and annually thereafter. Induction treatment consisted of IL-2 antibodies. We retrieved data on donor age and type (deceased or living), the number of human leukocyte antigen mismatches, and cold ischemia time from the EUROTRANSPLANT database, which was established in 1968 and holds complete entries of organ donor characteristics from transplants that have been performed in the EUROTRANSPLANT region to which Austria belongs [29]. Information on biopsy confirmed acute rejection defined according to Banff 97 criteria were extracted from the Vienna Kidney Biopsy Registry, which is composed of standardized descriptions of renal histopathology of native and transplant kidney biopsies [30].

All end-stage renal disease patients recorded in the OEDTR who received their first single-organ, ABO-compatible kidney transplant between January 1, 1990, and December 31, 2012, with an initial steroid-containing immunosuppressive regimen were included in this study and followed up until November 19, 2014.

The exposure of interest, ‘steroid withdrawal’, is a dichotomous time-dependent variable. Outcome variables were functional graft loss and all-cause death with functional graft. We performed cause-specific analyses of either event type. Graft survival time was defined as the time from transplantation until either permanent return to dialysis treatment or second transplantation, counting death or end of follow-up as censored observations. Patient survival time was defined as the time from first kidney transplantation until death, censored for graft loss, and end of follow-up.

### Statistical analyses

Continuous variables are expressed by mean and standard deviation, categorical variables are presented by frequencies and percentages.

To investigate the long-term effects of steroid withdrawal at various time points after kidney transplantation, we chose the landmarking approach, by which causal effects can be inferred under the usual assumptions of propensity score analyses [31]. Specific points in time following engraftment, so called landmark times, were pre-defined at 3-month intervals until 10 years after engraftment. At each of these landmark times, study participants were classified as either ‘steroid withdrawal’ or ‘steroid maintenance’ depending on steroid treatment status within the preceding time interval (first day after previous landmark time until current landmark time). Once patients were classified as ‘steroid withdrawal’ at a specific landmark time they were excluded from consideration at subsequent landmark times (Additional file 1: Figure S1).

Confounding by indication, caused by any potential difference in covariates between patients withdrawn from steroids and patients maintained on steroids that could have influenced the decision to withdraw or maintain steroids at a given landmark time, was addressed by introducing a landmark-time-dependent propensity score for matching steroid-maintenance patients to steroid-withdrawal patients at each landmark time [32–34]. First, we computed a logistic regression model to calculate the probability of steroid withdrawal or maintenance for each patient in the risk set at each landmark time based on the most recent values of confounding covariates (Additional file 1: Figure S2). As a second step, we matched patients withdrawn from steroids to patients maintained on steroids based on these individual propensity scores at each landmark time to generate a cohort of steroid withdrawal and steroid maintenance patients whose only remaining difference, in theory, is the steroid treatment status at a given landmark time. Using these matched study cohorts, we computed cause-specific cumulative incidence functions for the competing event type graft loss and death with functional graft and compared them between steroid treatment groups at specific landmark times. To summarize differences in graft loss and mortality following steroid withdrawal or maintenance at different time points, we estimated a landmark-stratified Cox supermodel using all matched study cohort data from all landmarks. In this supermodel, we included an interaction of steroid withdrawal status with landmark time, smoothing transitions between neighboring points in time using restricted cubic splines with knots at 1, 2, and 4 years [35–37]. This approach yielded the landmark-specific, propensity score-adjusted hazard ratios and 95% confidence intervals from which the time point with the largest benefit from discontinuation of steroids could be determined. Assessment of the proportional hazards assumption was conducted using a log minus log plot based on the cause-specific cumulative hazard estimated by the Kaplan–Meier method with weights according to the matching procedure. To deal with missing data in the covariates used for the propensity score, multiple imputation was employed [38, 39]. For steroid withdrawal status (the exposure of interest), no imputations were necessary. To determine whether biomarkers of cardiovascular risk improved after steroid withdrawal, we compared serum cholesterol, fasting glucose, the number of antihypertensive drugs, and body mass index before and after steroid withdrawal (Additional file 1).

A 95% confidence interval excluding parity or a two-sided *P* value less than 0.05 was considered as indication for statistical significance. For all analyses, the software R (version 3.2.1) was used. The study was approved by the Ethics Committee of the Medical University Vienna (1359/2014) and performed in accordance with the Declaration of Helsinki. The detailed statistical methods are outlined in Additional file 1.

## Results

### Patient characteristics at transplantation

We identified 6070 first kidney transplant recipients within the observation period in the Austrian Dialysis and Transplant Registry who met our inclusion criteria. We excluded 900 patients, because the initial immunosuppressive regimen did not contain steroids or entries were missing.

The baseline characteristics of the study cohort at transplantation and stratified by steroid treatment status 3 years after transplantation are listed in Table 1. At transplantation, the study cohort was 48 (±15) years old on average and most were men (64%); 94% of the patients received an immunosuppressive regimen based on a calcineurin inhibitor. The prevalence of diabetes was 27%, and 83% of the patients had arterial hypertension. Three years after transplantation, more patients in the steroid withdrawal group had diabetes mellitus compared to patients who were maintained on steroids at that time, suggesting a clinical indication (39% vs. 17%), but characteristics were otherwise similar between the two groups as well as compared with baseline at transplantation. After adjustment for this confounding by indication using propensity score-matched cohorts, the difference in diabetes between the two treatment groups was greatly reduced to 16% versus 13% (Table 2).

**Table 1:** Baseline characteristics of study participants at transplantation

Characteristics	n	At transplantation
Number of patients		5170
Recipient age, years (mean ± SD)	5170	48 (15)
Female recipients, n (%)	5170	1876 (36)
Diabetes mellitus, n (%)	3088	839 (27)
Arterial hypertension, n (%)	3217	2686 (83)
Living donor, n (%)	5116	645 (13)
Donor age, years (mean ± SD)	5108	46 (16)
Sum of human leukocyte antigen mismatch (mean ± SD)	4493	2.9 (1.3)
Immunosuppression, n (%)	5170	
Cyclosporine A-based regimen		2579 (50)
Tacrolimus-based regimen		2287 (44)
Other regimen		304 (6)

Continuous variables are described with mean and standard deviation and categorical variables with frequency and percentage

**Table 2:** Crude and matched characteristics of study participants 3 years after transplantation; 294 grafts were lost and 210 deaths occurred by 3 years after transplantation

	3 years after transplantation									
	Crude					Matched				
Characteristics	n	Steroid withdrawal	n	Steroid maintenance	SMD	n	Steroid withdrawal	n	Steroid maintenance	SMD
Number of patients		1272		2781			884		1203	
Recipient age, years (mean ± SD)	1272	49 (14)	2781	47 (16)	11%	884	48 (14)	1203	46 (16)	9%
Female recipients, n (%)	1272	495 (39)	2781	1012 (36)	5%	884	348 (39)	1203	457 (38)	3%
Diabetes mellitus, n (%)	750	290 (39)	1828	309 (17)	52%	465	74 (16)	750	100 (13)	7%
Arterial hypertension, n (%)	957	814 (85)	1922	1607 (84)	4%	655	543 (83)	810	675 (83)	2%
Living donor, n (%)	1266	180 (14)	2735	323 (12)	7%	880	117 (13)	1178	144 (12)	3%
Donor age, years (mean ± SD)	1261	43 (16)	2732	45 (16)	10%	877	44 (15)	1175	44 (16)	4%
Sum of human leukocyte antigen mismatch (mean ± SD)	1117	3.1 (1.4)	2435	2.6 (1.3)	38%	604	2.9 (1.3)	839	2.8 (1.3)	5%
Immunosuppression, n (%)	1272		2781			884		1203		
Cyclosporine A-based regimen		648 (51)		1673 (60)	19%		493 (56)		771 (64)	17%
Tacrolimus-based regimen		601 (47)		1004 (36)	23%		376 (43)		419 (35)	16%
Other regimen		23 (2)		104 (4)	11%		15 (2)		13 (1)	6%

Continuous variables are described with mean and standard deviation and categorical variables with frequency and percentage. Standardized mean difference (SMD) between steroid withdrawal and steroid maintenance groups were calculated for each covariate to quantify the difference between treatment groups. Time-dependent propensity score matching greatly reduced the difference in covariates between the two treatment groups

**Fig. 1:**
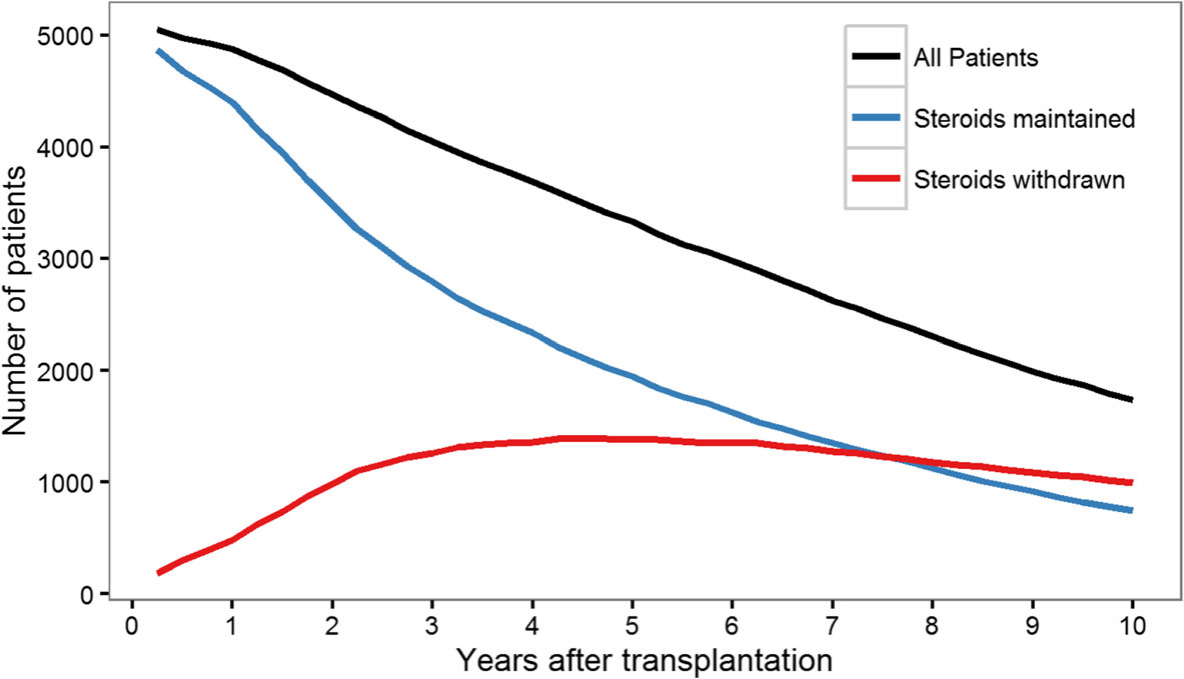
Shows the number of patients at risk, who were alive with a functioning graft throughout follow-up after transplantation in total and according to steroid treatment status

In total, 2142 patients were withdrawn from steroids within the study period (Fig. 1, Additional file 1: Figure S3). Median follow-up time was 6.5 years (25th percentile: 2.5 years; 75th percentile: 9.5 years). The fitted propensity score models to estimate the probability for steroid withdrawal at each landmark are shown in Additional file 1: Table S1, and reached a median concordance index of 0.68 (25th percentile: 0.61; 75th percentile: 0.71).

### Functional graft loss

**Fig. 2:**
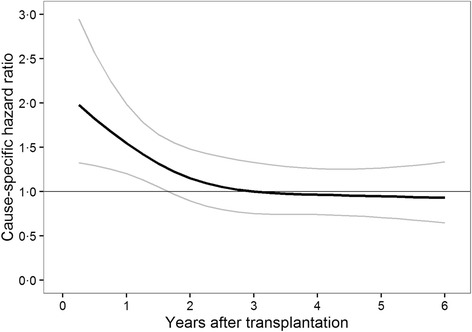
Shows hazard ratios and 95% confidence intervals for functional graft loss estimated from the Cox supermodel. The rate of graft loss was higher when steroids were withdrawn within the first 2 years after transplantation, while graft loss was unaffected by steroid withdrawal at later landmarks

Overall, 1131 patients in our study cohort lost their graft within the study period. In the landmark Cox supermodel the rate of graft loss was significantly higher for patients who were withdrawn from steroids within the first 18 months after transplantation compared to steroid maintenance during this time (6 months after transplantation: HR = 1.8; 95% CI, 1.3 to 2.6; 12 months after transplantation: HR = 1.6; 95% CI, 1.2 to 2.0; 18 months after transplantation: HR = 1.3; 95% CI, 1.1 to 1.6; 24 months after transplantation: HR = 1.2; 95% CI, 0.9 to 1.5; Fig. 2). This is supported by significantly more acute rejections in the withdrawal group if steroids were discontinued within the first 12 months after transplantation (17.6% vs. 7.2% within the first 6 months after transplantation, *P* < 0.001; 1.6% vs. 0.4% between 7 and 12 months after transplantation, *P* < 0.001; Table 3). Steroid withdrawal beyond 2 years after transplantation did not have an effect on graft loss (three years after transplantation: HR = 1.0, 95% CI, 0.8 to 1.3; 6 years after transplantation: HR = 0.9; 95% CI, 0.7 to 1.3).

**Table 3:** Frequency and percentage of biopsy-confirmed acute rejection within the first 6 months, between 7 and 12 months, between 13 and 24 months, and between 25 and 60 months after kidney transplantation are shown and compared between the two treatment groups, steroid withdrawal and steroid maintenance using a χ^2^ test

Time after transplantation	Steroid withdrawal, n (%)	Steroid maintenance, n (%)	*P* value
0–6 months	53 (17.6)	348 (7.2)	<0.001
7–12 months	8 (1.6)	16 (0.4)	<0.001
13–24 months	10 (1.0)	21 (0.5)	0.2
25–60 months	12 (0.7)	36 (1.3)	0.1

### All-cause mortality with functional graft

**Fig. 3:**
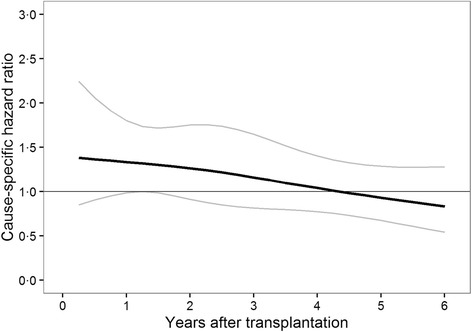
Shows hazard ratios and 95% confidence intervals for all-cause mortality with functional graft estimated from the Cox supermodel. The rate of death was not different between patients withdrawn from steroids compared to patients maintained on steroids at any landmark time

A total of 821 patients with a functional graft died within the study period, 303 due to cardiovascular causes, 208 as a result of infections, 122 due to malignancies, and 188 from other causes. We found no significant difference in all-cause mortality with functional graft between patients who were withdrawn from steroids and patients who were still receiving steroids at any landmark time in the landmark Cox supermodel (6 months after transplantation: HR = 1.4; 95% CI, 0.9 to 2.0; 1 year after transplantation: HR = 1.3; 95% CI, 1.0 to 1.8; 3 years after transplantation: HR = 1.2; 95% CI, 0.8 to 1.7; 6 years after transplantation: HR = 0.8; 95% CI, 0.5 to 1.3; Fig. 3).

For both outcomes, assessment of proportional hazards suggested some possible time-dependence of hazard ratios, yet with introduction of administrative censoring after 5 years, the estimated average hazard ratios were virtually unchanged compared to our main unrestricted analysis. Similarly, sensitivity analyses of our imputation approach indicated robust results (Additional file 1: Figure S4–Figure S7).

The comparisons of cardiovascular risk factors before and after steroid withdrawal showed that there was no difference in any of these cardiovascular surrogate outcomes (Additional file 1: Figure S8).

## Discussion

Our study is the first to evaluate various time points of steroid withdrawal following kidney transplantation using time point-specific propensity score matching and dynamic prediction by land-marking. Our results demonstrate that steroid withdrawal within the first 18 months after transplantation is associated with an increased rate of graft loss compared to steroid maintenance during that time, while mortality is unaffected by steroid withdrawal at any time point after transplantation compared to steroid maintenance.

Since a Canadian trial from the early 90s reported an increased rate of graft loss after steroid withdrawal compared to cyclosporine and steroids, more recent trials with contemporary immunosuppression consistently concluded that graft loss and mortality was not different between patients who were withdrawn from steroids compared to patients who received steroids, even if steroids were eliminated within the first days after engraftment [40–45]. It has been argued that IL-2 antibody induction and the combined use of tacrolimus and mycophenolate mofetil potentially facilitated safe steroid withdrawal [46]. Of note, the majority of these trials followed patients for up to 12 months only, and in light of this limited follow-up duration of rather small sample sizes, pooled analyses were likewise unable to show a difference in graft and patient survival following steroid withdrawal compared to steroid maintenance [11, 24]. Taking into account that approximately a third of the trials investigating steroid withdrawal inexplicably did not report these important outcomes, bias from selective outcome reporting must also be considered. Although clinical trials are the gold standard to investigate treatment effects, the currently available information from controlled data on long-term outcomes after steroid withdrawal is scarce. Besides this uncertainty, neither randomized trials, which can only investigate the effects of steroid withdrawal at one point in time, nor meta-analyses, which pool data of various time points into one analysis, are designed to determine an optimal timing for steroid withdrawal after kidney transplantation. Similarly, a large retrospective registry analysis from 2005 investigated steroid withdrawal in kidney transplant recipients but did not address the effect of different time points to withdraw steroids following transplantation [47]. The majority of trials investigated steroid withdrawal between 3 and 6 months after transplantation and outcomes following steroid withdrawal at later time points are particularly uncertain.

In agreement with others, we found that steroid withdrawal was associated with an increased risk of acute rejection, but in contrast to previous reports, our results revealed an increased rate of graft loss following steroid withdrawal within the first 18 months after transplantation compared to steroid maintenance during this time. Although an increased risk of acute rejection does not necessarily imply an increased rate of graft loss, we argue that the majority of trials were too small, with fewer than 300 participants, and too short, with a follow-up between 1 and 3 years, to determine long-term outcomes. It is not surprising that the rate of graft loss, as shown in our analysis, is higher if steroids are withdrawn within the first 18 months following engraftment as the immunological risk is higher earlier after transplantation. It is reasonable to assume that graft loss requires a larger amount of time to develop compared to acute rejection, which is an earlier outcome and is thus not observed in clinical trials. Our findings challenge current recommendations to abstain from steroid withdrawal in kidney transplantation beyond 1 week after engraftment, as well as proposals for safe steroid withdrawal between 3 and 6 months despite absence of long-term evidence from randomized trials [25].

In line with previous analyses, we found no significant difference in mortality between steroid withdrawal and maintenance at any time point after transplantation despite availability of long-term follow-up data in our registry. However, this does not only suggest that steroid withdrawal is not associated with an increased mortality but also that the desired survival benefit from discontinuation of long-term steroid maintenance might be absent. Although a statistically non-significant trend towards reduced mortality in patients who were withdrawn from steroids from 4 years after transplantation onwards can be discussed considering our results. A meta-analysis published in 2010 reported a reduction in cardiovascular risk, but analyses were based on surrogate outcomes rather than observed events of cardiovascular endpoints [48]. In contradiction, a review of the literature assessing long-term adverse effects of steroid treatment in rheumatic diseases reported no excess of cardiovascular disease [49]. The authors discussed that the overall fear of steroid-associated toxicity is probably overestimated in low dose long-term steroid treatment, but do acknowledge that additional risk factors, such as obesity, hypertension and diabetes, merit more careful observation of harmful side-effects associated with steroids. Although these findings in patients with rheumatic diseases might not be extrapolated to kidney transplant recipients, the absence of evidence on harmful effects of steroid withdrawal on mortality from controlled data combined with our findings justifies steroid withdrawal beyond 18 months after transplantation, as steroids beyond that time point are no longer required to protect the renal transplant.

When interpreting our study, some important limitations, in particular in relation to the retrospective nature of the collected data, need to be taken into account. Although we applied an advanced modelling approach with dynamic propensity score matching to address confounding by indication, our results may still be affected by unmeasured confounders which cannot be ruled out in any observational study [50]. Further, the choice of landmark time intervals potentially introduced survivor bias, namely that a patient has to survive until the next landmark time in order to be correctly classified and counted in the analysis, but should be non-differential between groups as this type of bias affects both steroid groups equally. However, the alternative to use shorter time intervals would have inflated the variance due to smaller sample sizes within the landmark-specific models. It has been recently discussed in the epidemiologic literature that a small non-differential bias should be preferred over inflation of variances in such circumstances [51]. Our study population is representative for a Central European, primarily Caucasian, population and results might thus not be generalizable to populations in other regions of the world or with different ethnic backgrounds.

Our study has a number of strengths. First and foremost, the availability of long-term outcome data of high quality in a well-maintained national registry with negligible numbers of patients lost to follow-up. Additionally, we have a wide range of available information that is periodically updated in the registry for multivariable adjustment. Furthermore, we have a large sample size of several thousand transplant recipients with a sufficiently large number of patients who were withdrawn from steroids to conduct adequate regression analyses, while the majority of existing trials included fewer than 300 participants [11]. Besides the available data itself, we meticulously computed landmark time-specific hazard ratios to determine the optimal time point for steroid withdrawal using contemporary statistical methods.

## Conclusions

In summary, randomized data do not provide conclusive information to inform clinical practice on steroid withdrawal in regard to long-term outcomes or optimal timing strategies for steroid withdrawal in kidney transplant recipients. Our study is the first to analyze multiple time points of steroid withdrawal with significant longer follow-up and suggests that the optimal time point for steroid withdrawal in kidney transplant recipients is beyond the first 18 months after transplantation.

## Additional file

Additional file 1: Figure S1. Schematic representation of the landmark Cox supermodel with dynamic propensity score-matched cohorts at each landmark. **Figure S2.** Schematic representation of the model building procedure. **Figure S3.** Patients represented in Fig. 1 are those withdrawn from steroids throughout the study period (2142 patients). Each bar represents 1 year after transplantation, and the height of the bar corresponds to the percentage of patients withdrawn from steroids within this timeframe. **Table S1.** The estimated odds ratios with 95% confidence intervals are given for the stratified propensity score model (averaged over all 60 imputed datasets). **Figure S4.** Shows plots of the cause-specific cumulative incidence function for functional graft loss (a) and all-cause mortality with functional graft (b) at specific landmark times after transplantation for the propensity score-matched study cohort (pooled over all 60 imputed datasets). **Figure S5.** Shows the log minus log plots for functional graft loss (a) and all-cause mortality with functional graft (b) at specific landmark times after transplantation for the propensity score-matched study cohort (pooled over all 60 imputed datasets). **Figure S6.** Shows the concatenated Cox supermodel for functional graft loss (a) and all-cause mortality with functional graft (b) with administrative restriction of follow-up at 5 years. By limiting follow-up duration in the presence of non-proportional hazards, it can be assessed whether results are sensitive to the proportionality of hazards. **Figure S7.** Shows results of the sensitivity analysis for functional graft loss (a) and all-cause mortality with functional graft (b) comparing results from our main analysis to those achieved by a complete case analysis and an analysis based on a restricted propensity score only using variables with less than 10% missing values. **Figure S8.** For each patient who was withdrawn from steroids within the study period (n = 2142), the difference in cardiovascular risk factors (the number of blood pressure medications, body mass index (BMI), serum cholesterol, fasting glucose) before and after steroid withdrawal were calculated. We first calculated the mean of all measurements within two landmarks before and after the time point of steroid withdrawal for each of the variables, and then calculated the difference in means. There was no difference in any of these surrogate cardiovascular outcomes, indicating that steroid withdrawal did not lead to an improvement of cardiovascular risk factors. (DOCX 451 kb)
