# Supplementary material for: Steroid withdrawal after renal transplantation: a retrospective cohort study
Source: BMC Med. 2017 Jan 12;15:8. doi: 10.1186/s12916-016-0772-6 (PMC5228116; doi:10.1186/s12916-016-0772-6)
Supplement: Additional file 1: Figure S1. — Schematic representation of the landmark Cox supermodel with dynamic propensity score-matched cohorts at each landmark. Figure S2. Schematic representation of the model building procedure. Figure S3. Patients represented in Fig. 1 are those withdrawn from steroids throughout the study period (2142 patients). Each bar represents 1 year after transplantation, and the height of the bar corresponds to the percentage of patients withdrawn from steroids within this timeframe. Table S1. The estimated odds ratios with 95% confidence intervals are given for the stratified propensity score model (averaged over all 60 imputed datasets). Figure S4. Shows plots of the cause-specific cumulative incidence function for functional graft loss (a) and all-cause mortality with functional graft (b) at specific landmark times after transplantation for the propensity score-matched study cohort (pooled over all 60 imputed datasets). Figure S5. Shows the log minus log plots for functional graft loss (a) and all-cause mortality with functional graft (b) at specific landmark times after transplantation for the propensity score-matched study cohort (pooled over all 60 imputed datasets). Figure S6. Shows the concatenated Cox supermodel for functional graft loss (a) and all-cause mortality with functional graft (b) with administrative restriction of follow-up at 5 years. By limiting follow-up duration in the presence of non-proportional hazards, it can be assessed whether results are sensitive to the proportionality of hazards. Figure S7. Shows results of the sensitivity analysis for functional graft loss (a) and all-cause mortality with functional graft (b) comparing results from our main analysis to those achieved by a complete case analysis and an analysis based on a restricted propensity score only using variables with less than 10% missing values. Figure S8. For each patient who was withdrawn from steroids within the study period (n = 2142), the difference in cardiovascular risk factors (th [file 12916_2016_772_MOESM1_ESM.docx]

Additional file 1

**Steroid withdrawal after renal transplantation: a retrospective cohort study**

## Author list

Maria C Haller, MD^1,2,3^, Michael Kammer, DI^1^, Alexander Kainz, PhD^4^, Heather J Baer, ScD^,7,8^ , Georg Heinze, PhD^1^ and Rainer Oberbauer MD^4,5^

## Author’s affiliations

1. Center for Medical Statistics, Informatics and Intelligent Systems (CeMSIIS), Section for Clinical Biometrics, Medical University of Vienna, Vienna, Austria
2. Department for Internal Medicine III, Nephrology and Hypertension Diseases, Transplantation Medicine and Rheumatology, Krankenhaus Elisabethinen, Linz, Austria
3. Methods Support Team ERBP, Ghent University Hospital, Ghent, Belgium
4. Department of Nephrology, Medical University of Vienna, Vienna, Austria
5. Austrian Dialysis and Transplant Registry, Austria
6. Division of General Internal Medicine and Primary Care, Brigham and Women’s Hospital, Boston, MA
7. Department of Medicine, Harvard Medical School, Boston, MA
8. Department of Epidemiology, Harvard T.H. Chan School of Public Health, Boston, MA

**Table of Contents**

[Author list 1](#_Toc468449786)

[Author’s affiliations 1](#_Toc468449787)

[**Extended statistical methods** 3](#_Toc468449788)

[**Figure S1** 6](#_Toc468449789)

[**Figure S2** 6](#_Toc468449790)

[**Figure S3** 7](#_Toc468449791)

[**Table S1** 8](#_Toc468449792)

[**Figure S4** 10](#_Toc468449793)

[**Figure S4a** 10](#_Toc468449794)

[**Figure S4b** 10](#_Toc468449795)

[**Figure S5** 11](#_Toc468449796)

[**Figure S5a** 11](#_Toc468449797)

[**Figure S5b** 11](#_Toc468449798)

[**Figure S6** 12](#_Toc468449799)

[**Figure S6a** 12](#_Toc468449800)

[**Figure S6b** 13](#_Toc468449801)

[**Figure S7** 14](#_Toc468449802)

[**Figure S7a** 14](#_Toc468449803)

[**Figure S7b** 14](#_Toc468449804)

[**Figure S8** 15](#_Toc468449805)

[**References** 16](#_Toc468449806)

# **Extended statistical methods**

Continuous variables are expressed by mean and standard deviation, categorical variables are presented by frequencies and percentages.

We chose the landmarking approach for analysis, by which the causal effect of steroid withdrawal at various time points after transplantation can be inferred under the usual assumptions of propensity score analyses ([1](#_ENREF_1)) Specific points in time following engraftment, so called landmark times, were pre-defined at three month intervals until ten years after engraftment. At each of these landmark times, study participants were classified as either ‘steroid withdrawal’ or ‘steroid maintenance’ depending on steroid treatment status within the preceding time interval (first day after previous landmark time until current landmark time). Once patients were classified as ‘steroid withdrawal’ at a specific landmark time they were excluded from the risk sets corresponding to subsequent landmark times.

Confounding by indication was addressed by introducing a landmark-time-dependent propensity score for matching steroid-maintenance patients to steroid-withdrawal patients at each landmark time ([2](#_ENREF_2), [3](#_ENREF_3)). A caliper of 0.1 standard deviations of the logit of the propensity score was used as threshold for candidate matches and the two to three best matches were selected to minimize random variation of the matching ([4](#_ENREF_4)). The following confounding variables were selected based on clinical judgment and entered the logistic regression model for calculation of the propensity scores with their most recent value preceding the respective landmark time: donor type (deceased donor versus living donor) and age, HLA mismatch sum, recipient age at transplantation, recipient sex, primary renal diagnosis, co-morbidities (diabetes mellitus, arterial hypertension, coronary artery disease, chronic heart failure, chronic liver disease, chronic pulmonary disease), immunosuppression (Cyclosporine A based regimen, Tacrolimus based regimen, other), and serum creatinine ([5](#_ENREF_5)). Including any of the other available covariates from the database, such as proteinuria, panel reactive antibodies, serum cholesterol, blood glucose, in the models did not improve the propensity score as determined by the c-index and were therefore removed from the models for reasons of parsimony.

The model was then stratified by landmark time and a ridge penalty was employed to minimize problems due to small sample sizes at certain landmark times. Using this matched study cohort, we computed cause-specific cumulative incidence functions to compare graft loss and death with functional graft between steroid treatment groups at specific landmark times. Both event types were treated as competing events. Assessment of the proportional hazards assumption was conducted using a log minus log plot based on the cause-specific cumulative hazard estimated by the Kaplan-Meier method with weights according to the matching procedure.

To obtain landmark-specific, propensity score adjusted hazard ratios and 95% confidence intervals, we estimated an interaction of steroid withdrawal status with landmark time in a Cox supermodel. Landmark time was modeled using restricted cubic splines with knots at one, two and four years to smooth transitions between neighboring points in time ([6](#_ENREF_6), [7](#_ENREF_7)). With this approach the difference in graft loss and mortality between steroid withdrawal patients and steroid maintenance patients can be estimated for each landmark time and thus the time point with the largest benefit from discontinuation of steroids can be identified. Since patients may appear in the model at different landmark time points, we employed a robust covariance matrix to estimate the 95% confidence intervals for hazard ratios. To deal with missing data in the covariates used for the propensity score, multiple imputation by chained equations was used ([8](#_ENREF_8), [9](#_ENREF_9)). All 60 imputed versions of the dataset were analyzed separately and results were then combined following Rubin’s rules. Adequacy of the multiple imputation procedure was assessed by a comparison of results with the complete case analysis. In addition, we performed another sensitivity analysis reducing the set of adjustment variables to those variables which were available at baseline and for which less than 10% of the values were missing. For steroid withdrawal status, the exposure of interest, no imputation was necessary.

A two-sided p-value less than 0.05 was considered as indication for statistical significance. For all analyses the software R (version 3.2.1) was used. The study was approved by the Ethics Committee of the Medical University Vienna (1359/2014) and performed in accordance with the Declaration of Helsinki.

# **Figure S1**

Schematic representation of the landmark Cox supermodel with dynamic propensity score matched cohorts at each landmark.


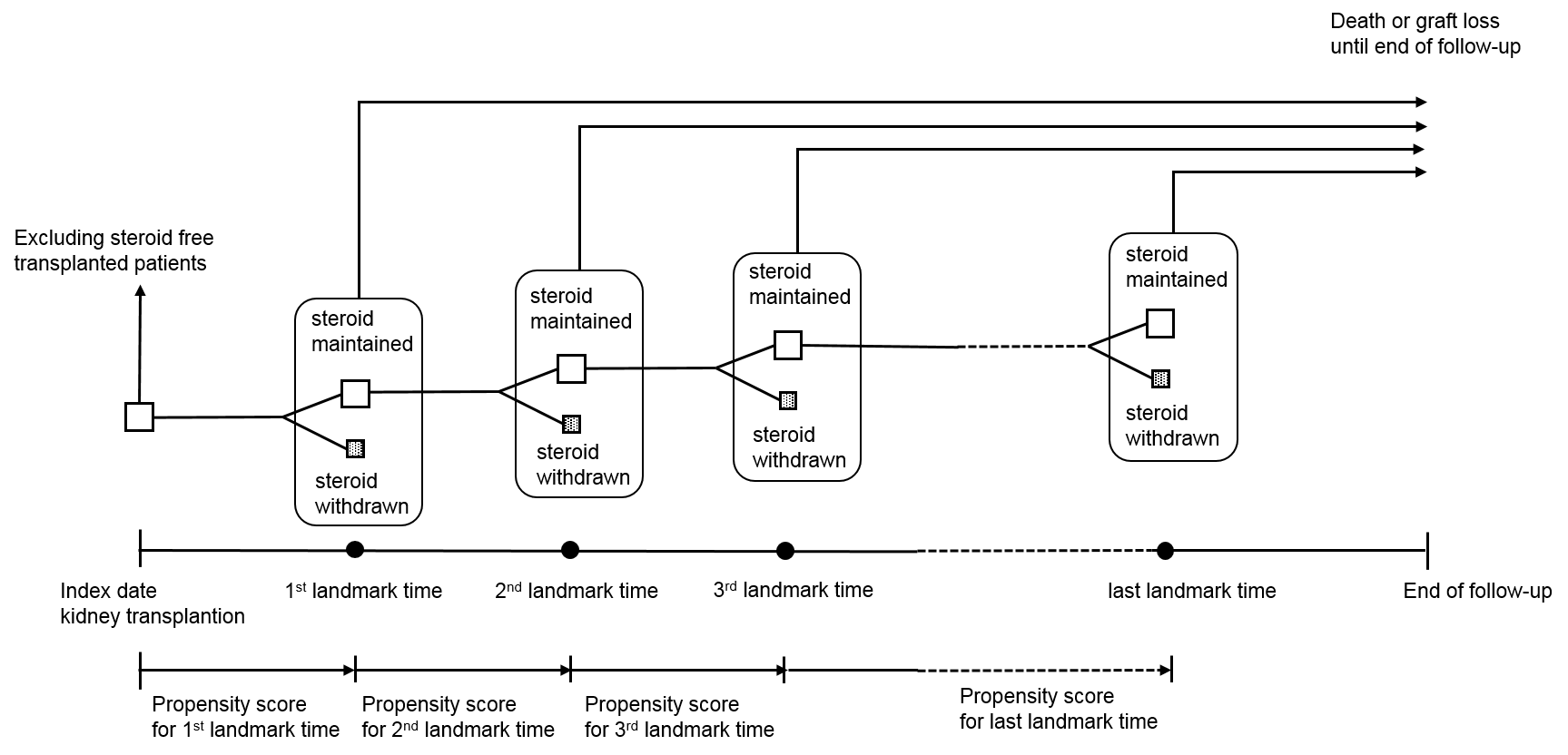


# **Figure S2**

Schematic representation of the model building procedure.


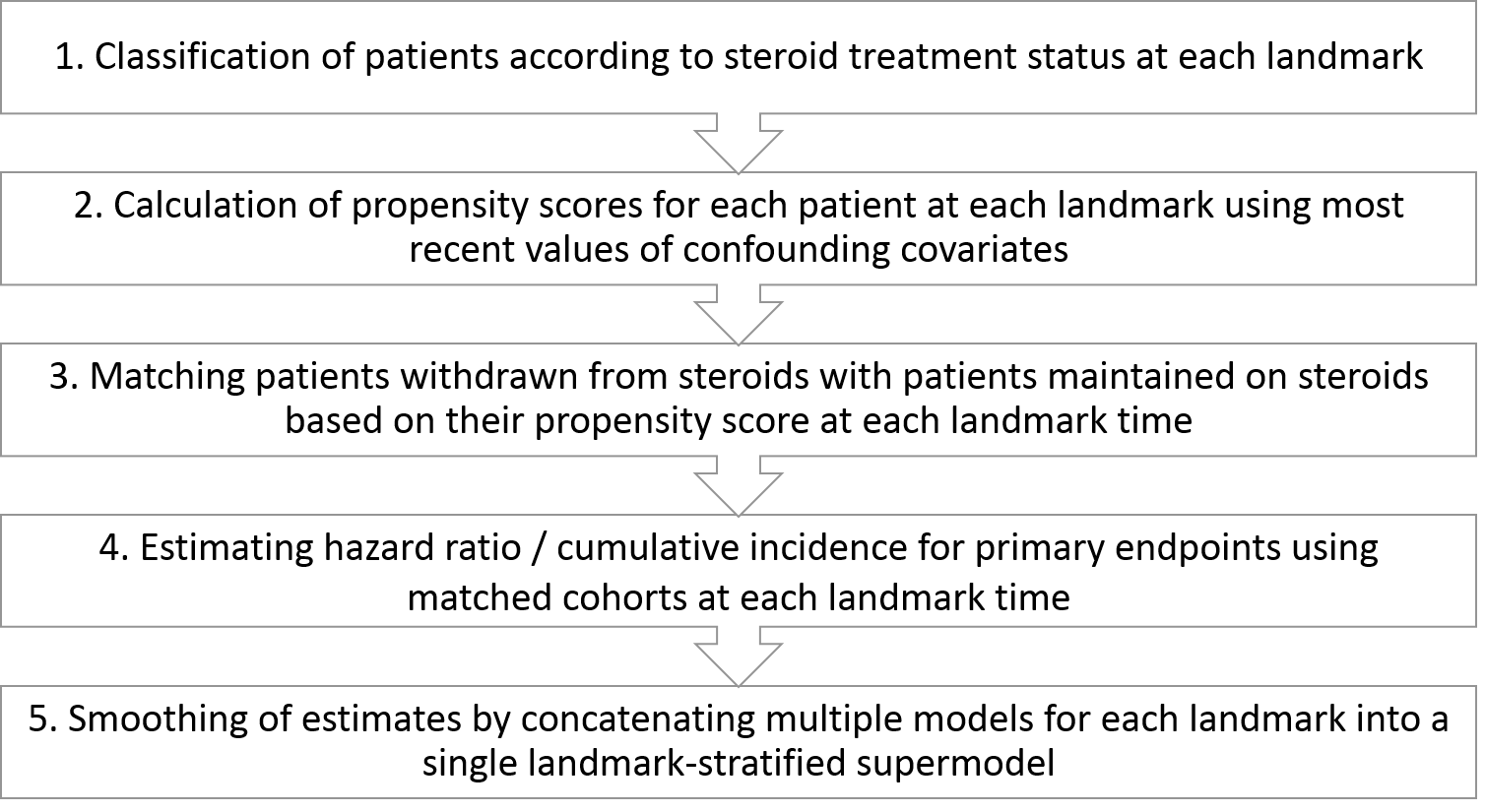


# **Figure S3**

Patients represented in figure 1 are those withdrawn from steroids throughout the study period (2142 patients). Each bar represents one year after transplantation, and the height of the bar corresponds to the percentage of patients withdrawn from steroids within this timeframe.


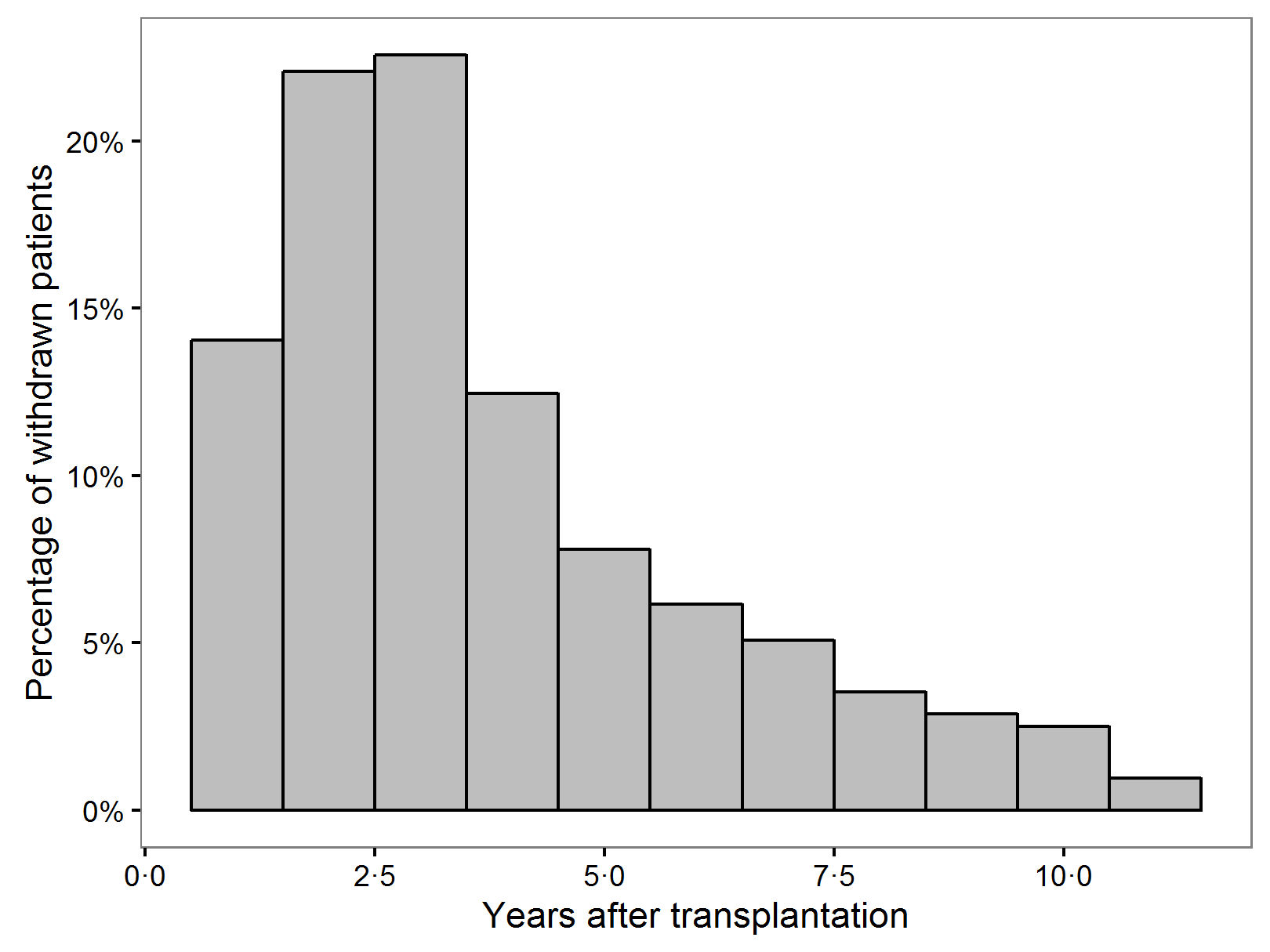


# **Table S1**

The estimated odds ratios with 95% confidence intervals are given for the stratified propensity score model (averaged over all 60 imputed datasets).

|  |  | **95% Confidence interval** | |
| --- | --- | --- | --- |
| **Variable** | **Odds ratio** | **Lower bound** | **Upper bound** |
| Intercept | 0.03 | 0.02 | 0.04 |
| Landmarktime (6 vs 3 months) | 0.65 | 0.51 | 0.82 |
| Landmarktime (9 vs 3 months) | 0.56 | 0.43 | 0.71 |
| Landmarktime (12 vs 3 months) | 0.64 | 0.51 | 0.82 |
| Landmarktime (15 vs 3 months) | 0.87 | 0.70 | 1.09 |
| Landmarktime (18 vs 3 months) | 0.85 | 0.68 | 1.08 |
| Landmarktime (21 vs 3 months) | 1.03 | 0.82 | 1.28 |
| Landmarktime (24 vs 3 months) | 1.02 | 0.82 | 1.28 |
| Landmarktime (27 vs 3 months) | 1.03 | 0.82 | 1.30 |
| Landmarktime (30 vs 3 months) | 0.64 | 0.49 | 0.84 |
| Landmarktime (33 vs 3 months) | 0.75 | 0.58 | 0.98 |
| Landmarktime (36 vs 3 months) | 0.68 | 0.52 | 0.90 |
| Landmarktime (39 vs 3 months) | 0.67 | 0.50 | 0.89 |
| Landmarktime (42 vs 3 months) | 0.54 | 0.39 | 0.74 |
| Landmarktime (45 vs 3 months) | 0.43 | 0.30 | 0.61 |
| Landmarktime (48 vs 3 months) | 0.64 | 0.47 | 0.87 |
| Landmarktime (51 vs 3 months) | 0.52 | 0.37 | 0.73 |
| Landmarktime (54 vs 3 months) | 0.42 | 0.28 | 0.61 |
| Landmarktime (57 vs 3 months) | 0.48 | 0.33 | 0.69 |
| Landmarktime (60 vs 3 months) | 0.40 | 0.27 | 0.60 |
| Landmarktime (63 vs 3 months) | 0.59 | 0.41 | 0.83 |
| Landmarktime (66 vs 3 months) | 0.46 | 0.31 | 0.68 |
| Landmarktime (69 vs 3 months) | 0.39 | 0.26 | 0.60 |
| Landmarktime (72 vs 3 months) | 0.66 | 0.46 | 0.94 |
| Landmarktime (75 vs 3 months) | 0.58 | 0.40 | 0.86 |
| Landmarktime (78 vs 3 months) | 0.27 | 0.15 | 0.46 |
| Landmarktime (81 vs 3 months) | 0.46 | 0.30 | 0.71 |
| Landmarktime (84 vs 3 months) | 0.42 | 0.26 | 0.67 |
| Landmarktime (87 vs 3 months) | 0.37 | 0.23 | 0.62 |
| Landmarktime (90 vs 3 months) | 0.34 | 0.20 | 0.58 |
| Landmarktime (93 vs 3 months) | 0.24 | 0.13 | 0.45 |
| Landmarktime (96 vs 3 months) | 0.48 | 0.30 | 0.77 |
| Landmarktime (99 vs 3 months) | 0.43 | 0.25 | 0.72 |
| Landmarktime (102 vs 3 months) | 0.45 | 0.27 | 0.76 |
| Landmarktime (105 vs 3 months) | 0.21 | 0.10 | 0.44 |
| Landmarktime (108 vs 3 months) | 0.44 | 0.25 | 0.76 |
| Landmarktime (111 vs 3 months) | 0.56 | 0.34 | 0.93 |
| Landmarktime (114 vs 3 months) | 0.52 | 0.31 | 0.89 |
| Landmarktime (117 vs 3 months) | 0.33 | 0.17 | 0.65 |
| Landmarktime (120 vs 3 months) | 0.43 | 0.23 | 0.79 |
| Mismatch sum (continuous) | 1.10 | 1.07 | 1.13 |
| Living donor vs deceased donor | 1.13 | 0.99 | 1.28 |
| Donor age (years, continuous) | 1.00 | 1.00 | 1.00 |
| Female vs male | 1.08 | 0.99 | 1.18 |
| Diabetic nephropathy vs else | 0.88 | 0.71 | 1.09 |
| Vascular nephropathy vs else | 0.75 | 0.59 | 0.95 |
| Glomerulonephritis vs else | 0.74 | 0.65 | 0.85 |
| Recipient age at transplantation (years, continuous) | 1.00 | 1.00 | 1.00 |
| Other vs Cyclosporine based immunosuppression | 0.49 | 0.37 | 0.65 |
| Tacrolimus vs Cyclosporine based immunosuppression | 0.97 | 0.88 | 1.06 |
| Diabetes mellitus vs none | 1.52 | 1.35 | 1.72 |
| Malignancy vs none | 0.88 | 0.72 | 1.08 |
| Chronic liver disease vs none | 1.16 | 0.99 | 1.36 |
| Chronic lung disease vs none | 0.91 | 0.75 | 1.10 |
| Chronic heart disease vs none | 1.09 | 0.98 | 1.21 |
| Number of antihypertensives (continuous) | 0.98 | 0.95 | 1.01 |
| Serum-Creatinin (mg/dl, continuous) | 0.95 | 0.91 | 1.00 |

# **Figure S4**

Shows plots of the cause-specific cumulative incidence function for functional graft loss (a) and all-cause mortality with functional graft (b) at specific landmark times after transplantation for the propensity score matched study cohort (pooled over all 60 imputed datasets).

# **Figure S4a**


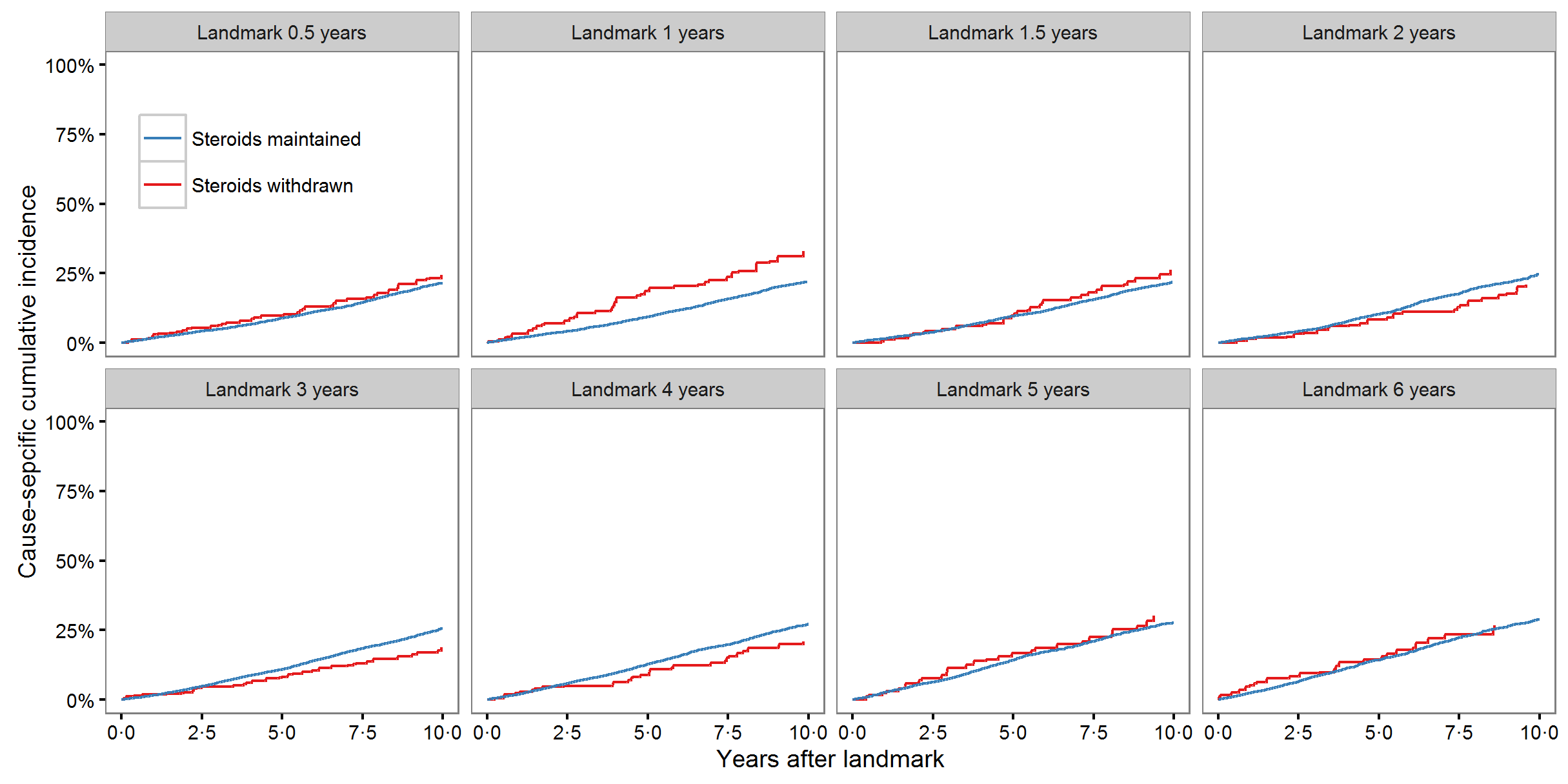


# **Figure S4b**


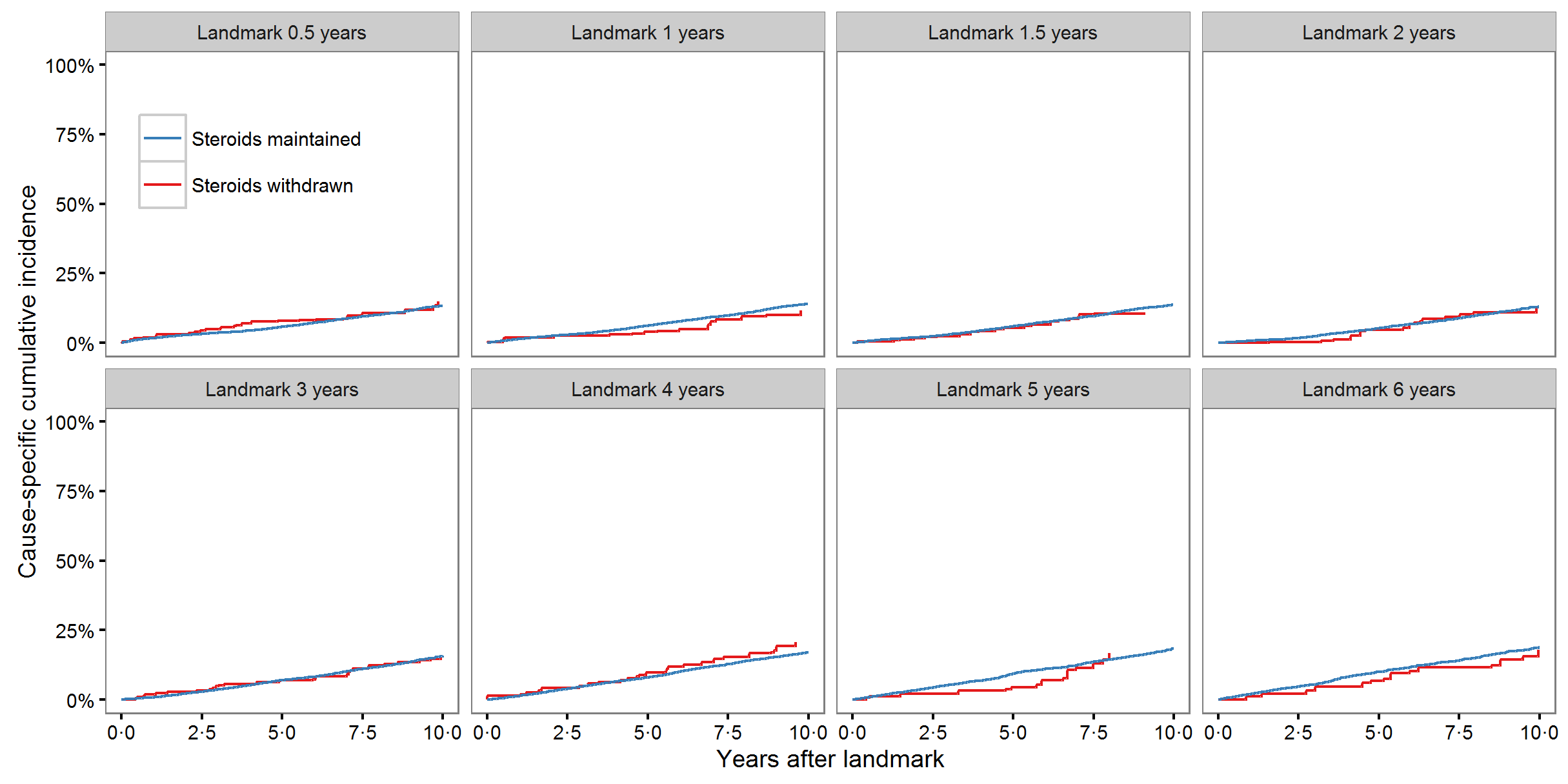


# **Figure S5**

Shows the log minus log plots for functional graft loss (a) and all-cause mortality with functional graft (b) at specific landmark times after transplantation for the propensity score matched study cohort (pooled over all 60 imputed datasets).

# **Figure S5a**


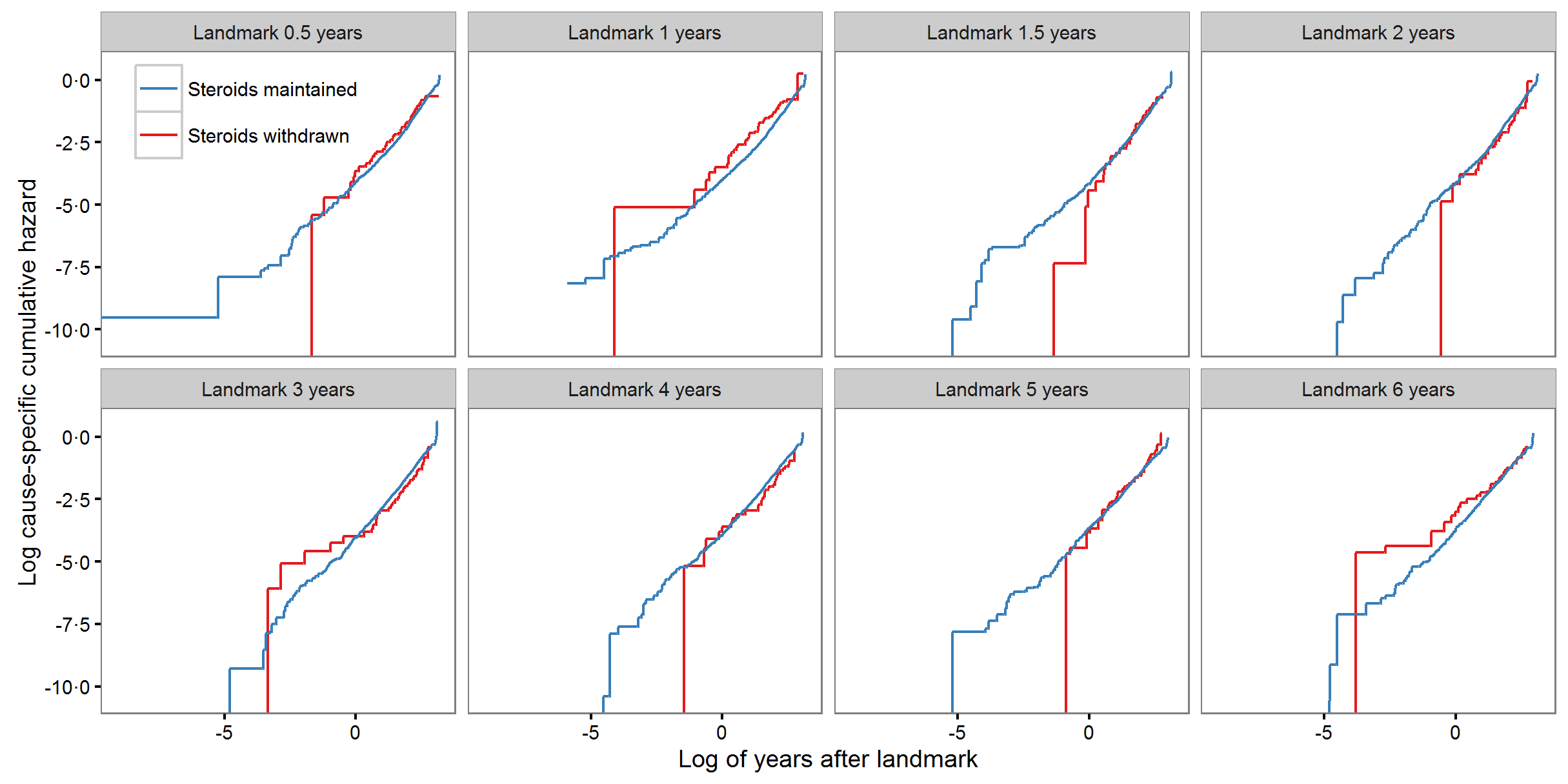


# **Figure S5b**


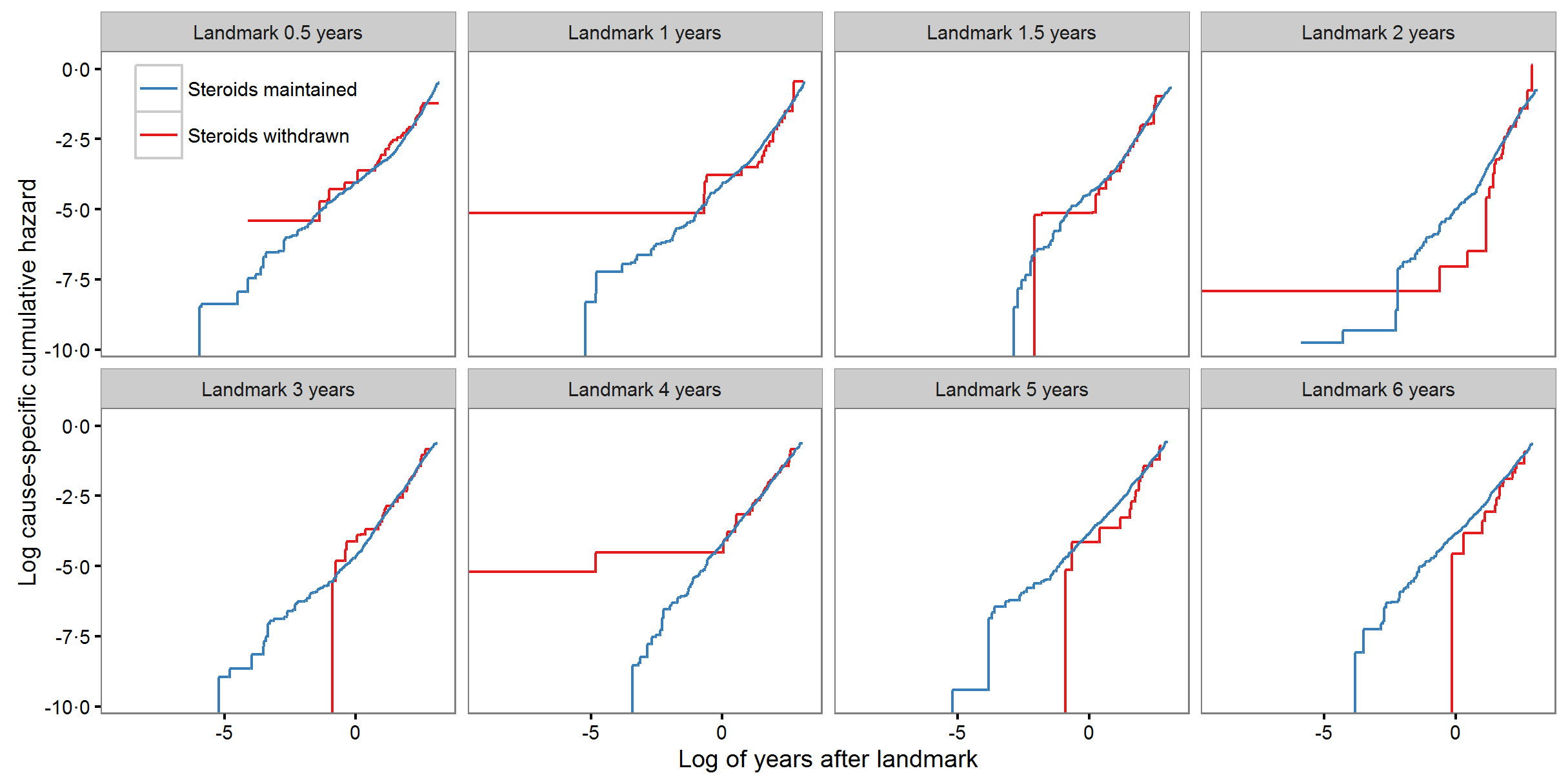


**Figure S6**

Shows the concatenated Cox supermodel for functional graft loss (a) and all-cause mortality with functional graft (b) with administrative restriction of follow-up at five years. By limiting follow-up duration in the presence of non-proportional hazards, it can be assessed whether results are sensitive to the proportionality of hazards.

# **Figure S6a**


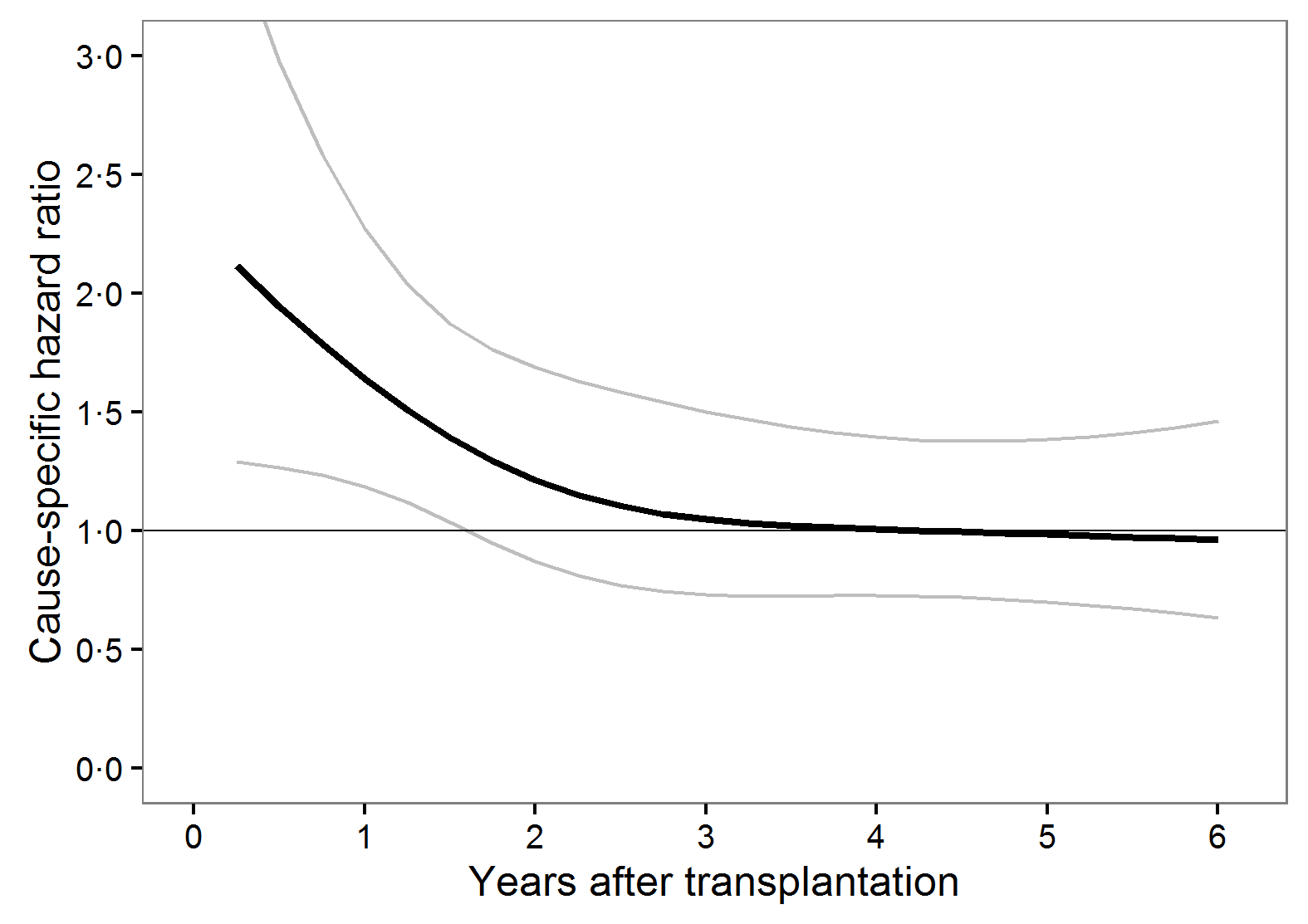


| **landmark time after transplantation** | **HR (95% CI)** |
| --- | --- |
| 6 months | 1·9 (1·3, 3·0) |
| 1 year | 1·6 (1·2, 2·3) |
| 3 years | 1.1 (0·7, 1·5) |
| 6 years | 1·0 (0·6, 1·5) |

# **Figure S6b**


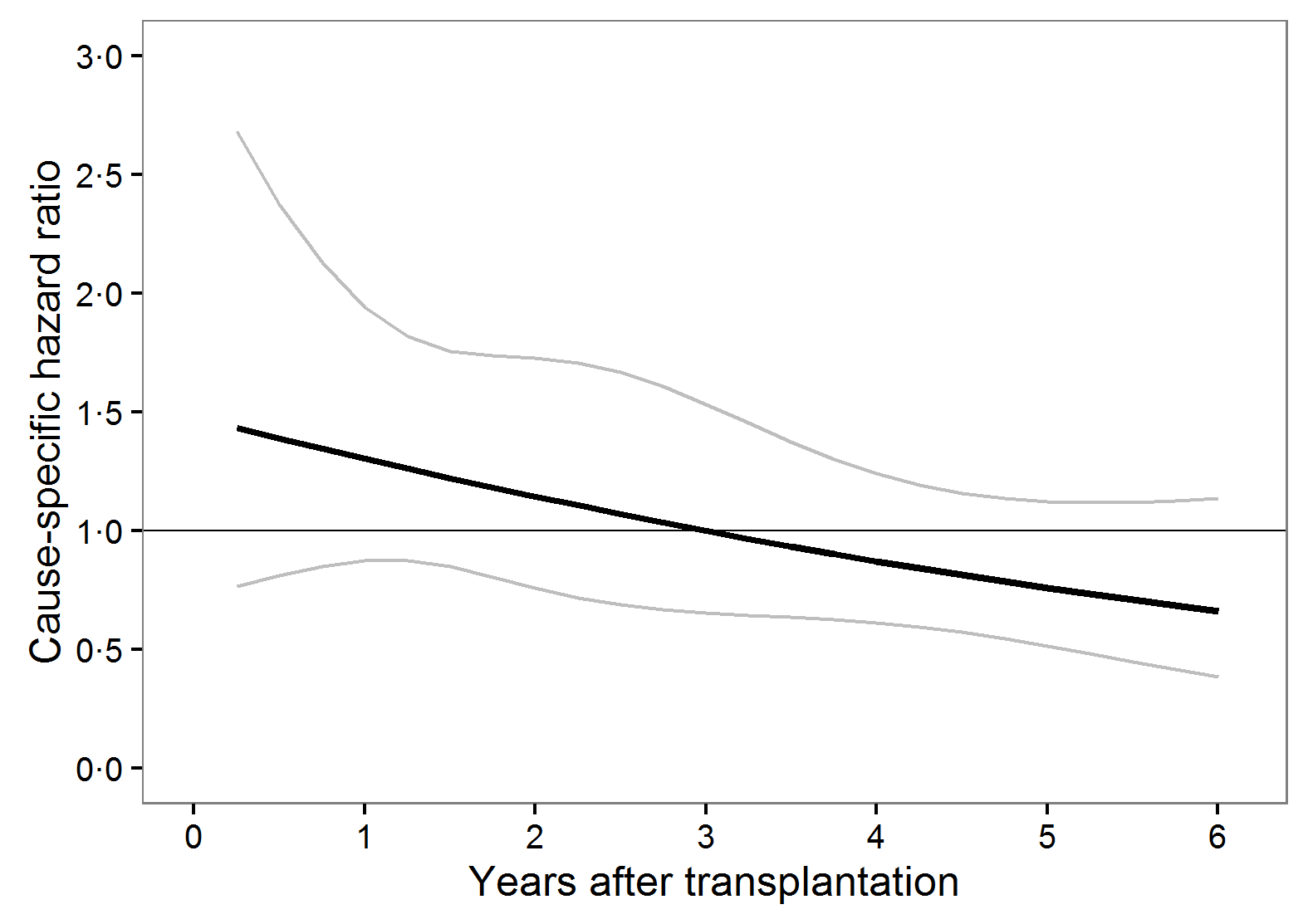


| **landmark time after transplantation** | **HR (95% CI)** |
| --- | --- |
| 6 months | 1·4 (0·8, 2·4) |
| 1 year | 1·3 (0·9, 1·9) |
| 3 years | 1·0 (0·7, 1·5) |
| 6 years | 0·7 (0·4, 1·1) |

**Figure S7**

Shows results of the sensitivity analysis for functional graft loss (a) and all-cause mortality with functional graft (b) comparing results from our main analysis to those achieved by a complete case analysis and an analysis based on a restricted propensity score only using variables with less than 10% missing values.

**Figure S7a**


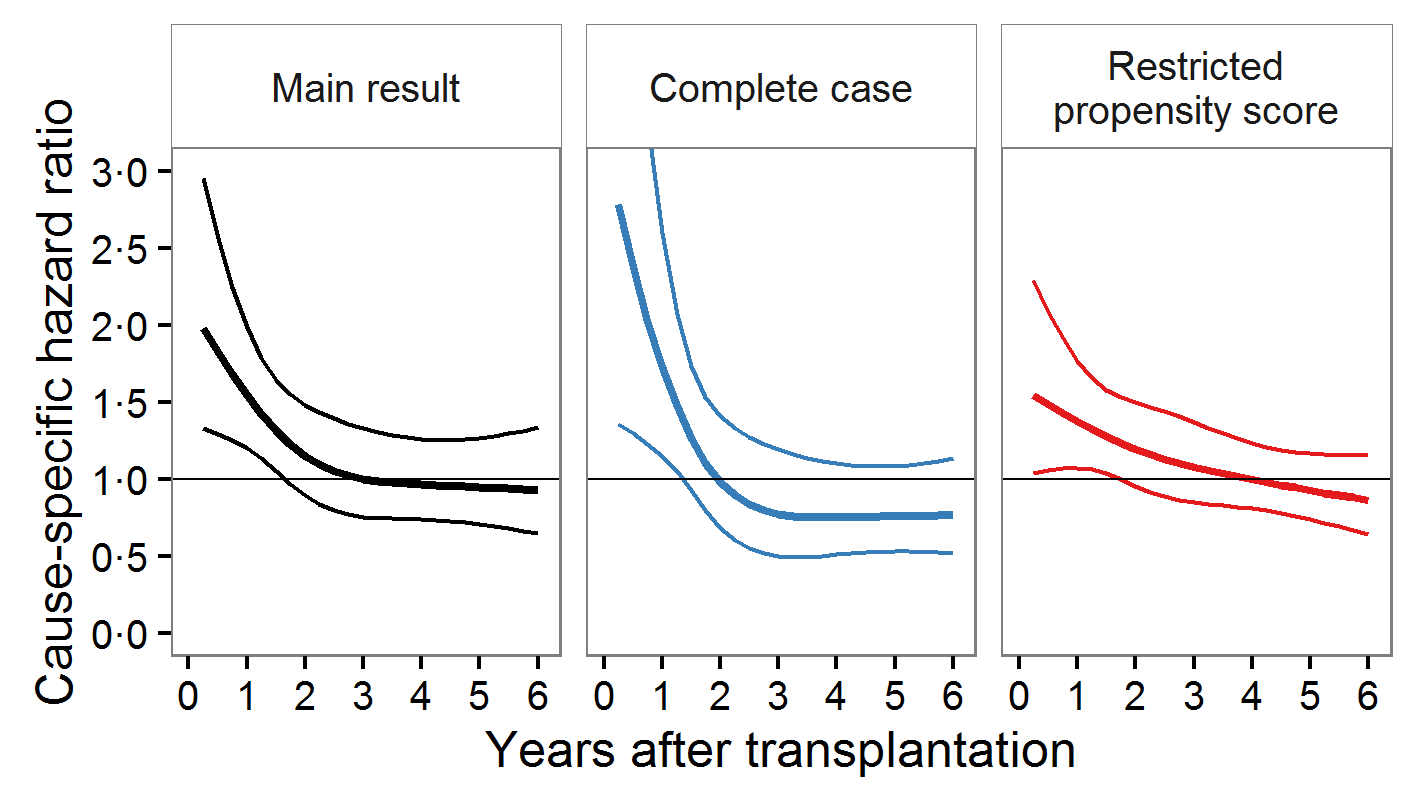


**Figure S7b**


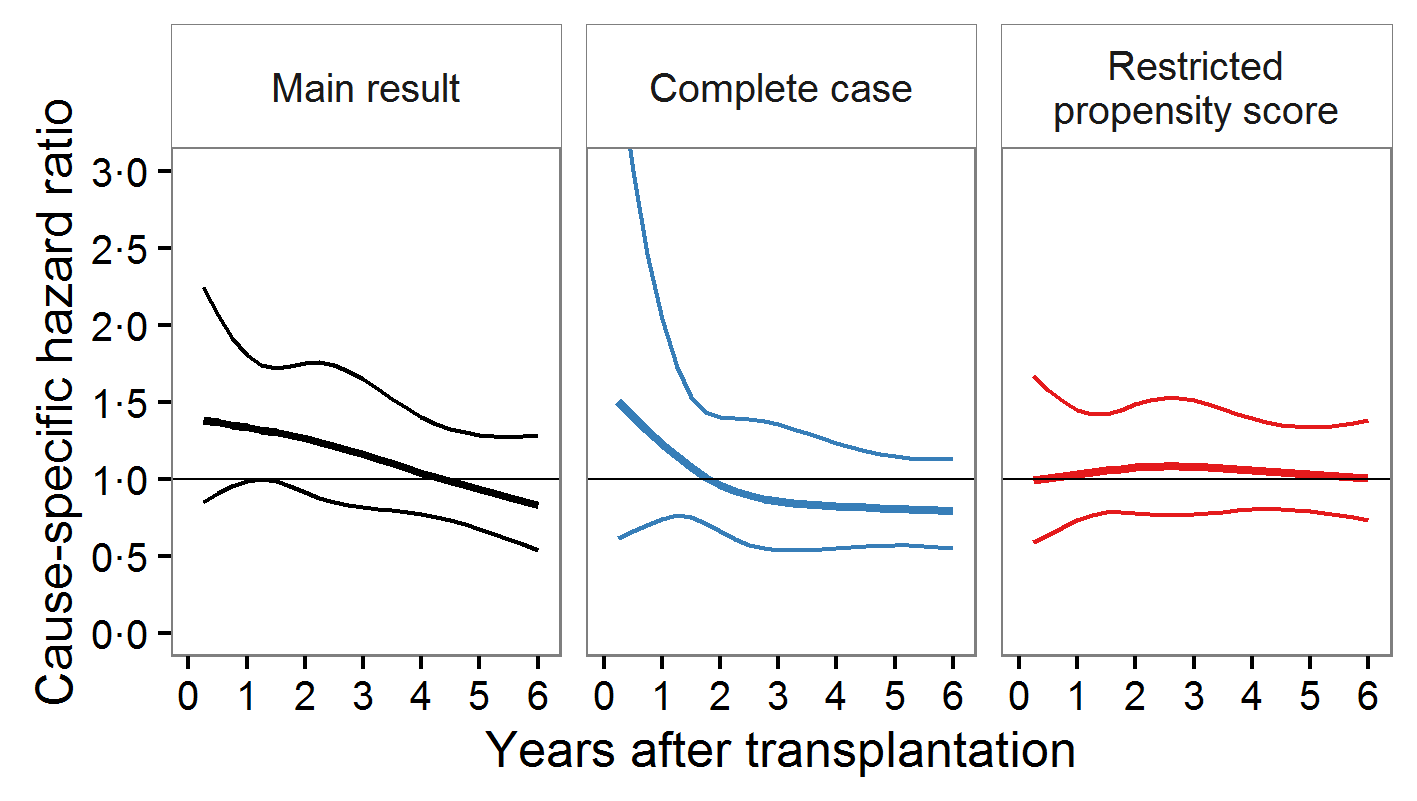


**Figure S8**

For each patient who was withdrawn from steroids within the study period (n=2142), the difference in cardiovascular risk factors (the number of blood pressure medications, body mass index (BMI), serum cholesterol, fasting glucose) before and after steroid withdrawal were calculated. We first calculated the mean of all measurements within 2 landmarks before and after the time point of steroid withdrawal for each of the variables, and then calculated the difference in means. There was no difference in any of these surrogate cardiovascular outcomes, indicating that steroid withdrawal did not lead to an improvement of cardiovascular risk factors.


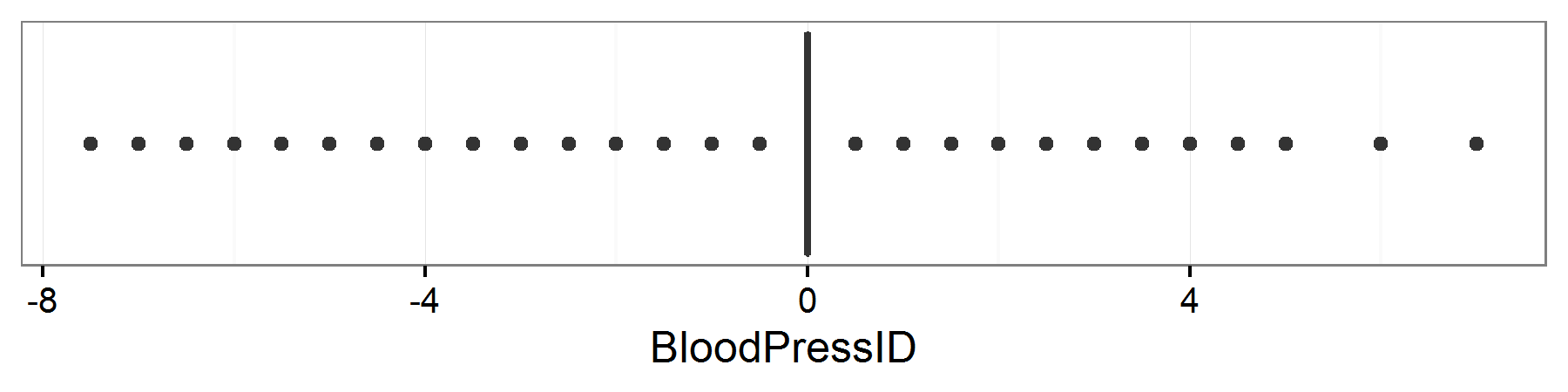

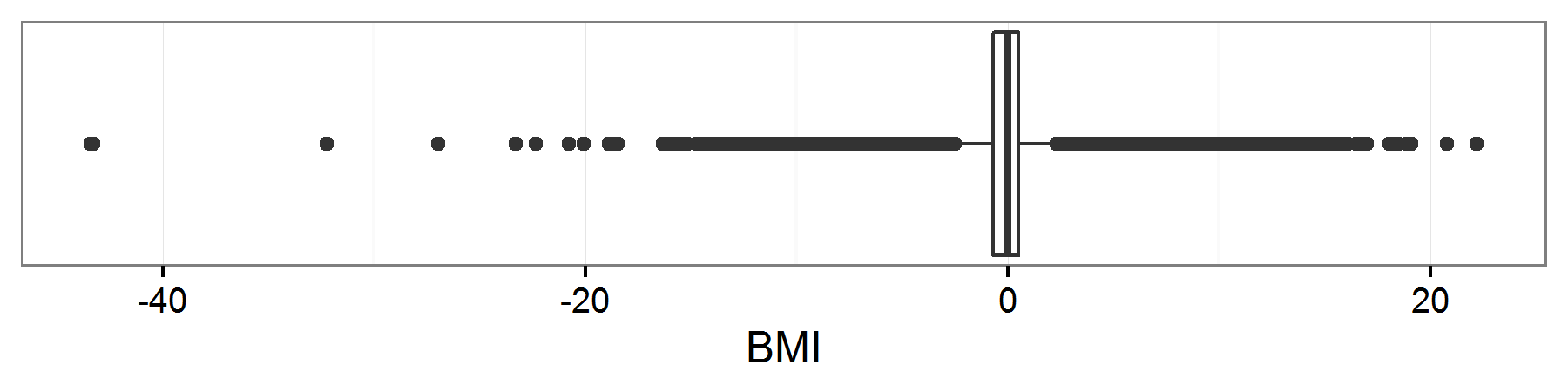

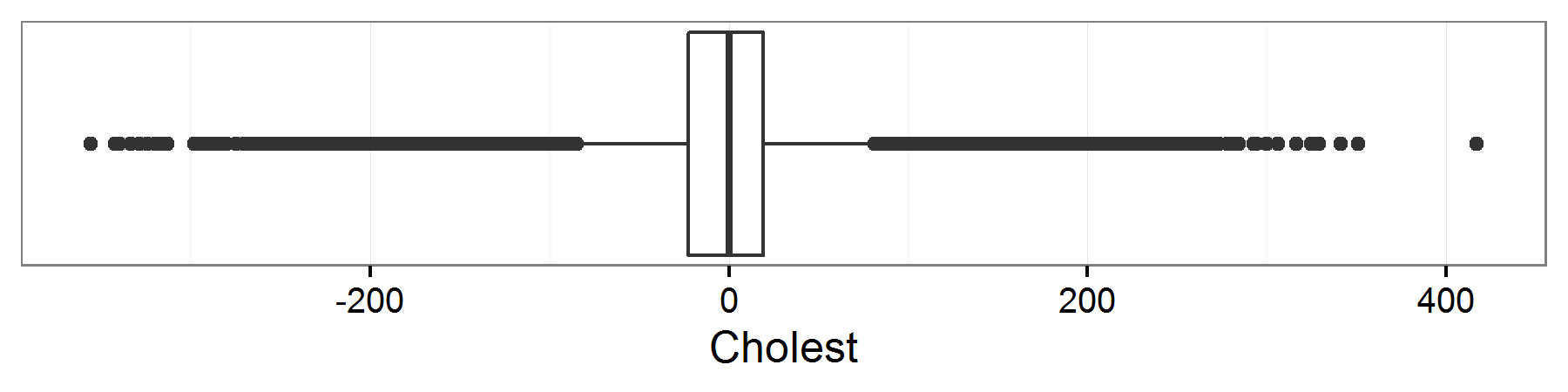

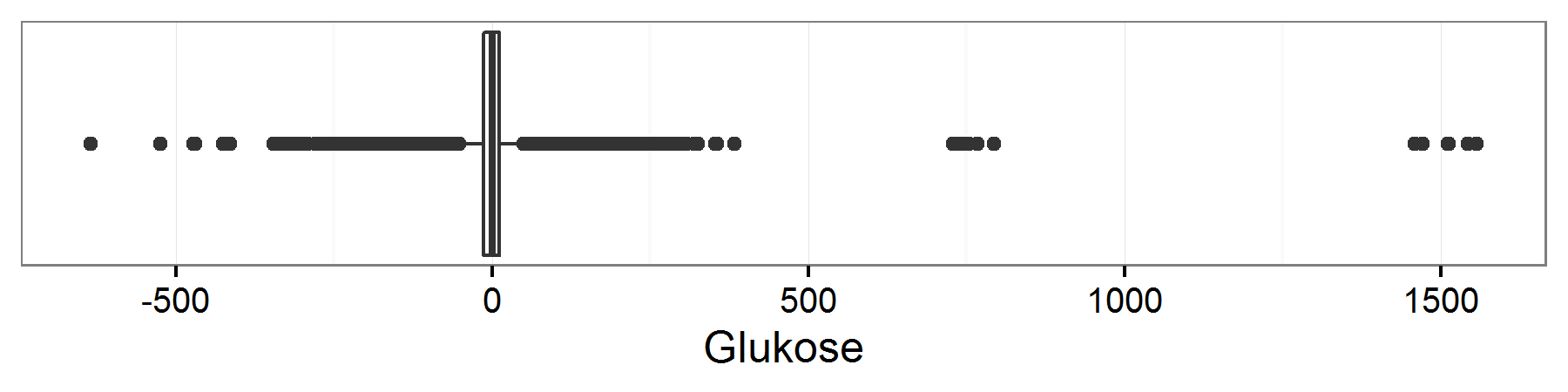


# **References**

1. Heinze G, Juni P. An overview of the objectives of and the approaches to propensity score analyses. Eur Heart J. 2011 Jul;32(14):1704-8. doi: 10.093/eurheartj/ehr031. Epub 2011 Feb 28.

2. Hernan MA, Robins JM. Estimating causal effects from epidemiological data. J Epidemiol Community Health. 2006 Jul;60(7):578-86.

3. Rosenbaum P, Rubin D. The central role of the propensity score in observational studies for causal effects. . Biometrika 1983;70:41 –55.

4. Sekhon JS. Multivariate and Propensity Score Matching Software with Automated Balance Optimization: The Matching Package for R. Journal of Statistical Software. 2011;42(7):1-52.

5. Harrel F. Regression Modeling Strategies: With Applications to Linear Models, Logistic Regression and Survival Analysis. . New York, NY: Springer Inc; . 2001.

6. Anderson JR, Cain KC, Gelber RD. Analysis of survival by tumor response and other comparisons of time-to-event by outcome variables. J Clin Oncol. 2008;26(24):3913-5.

7. Van Houwelingen HC. Dynamic Prediction by Landmarking in Event History Analysis. Scandinavian Journal of Statistics. 2007;34:70–85.

8. Little R, Rubin D. Statistical analysis with missing data. 2nd ed: New York, Wiley; 2002.

9. van Buuren S, Groothuis-Oudshoorn K. mice: Multivariate Imputation by Chained Equations in R. Journal of Statistical Software. 2011;45(3):1-67.
